# TAMing pancreatic cancer: combat with a double edged sword

**DOI:** 10.1186/s12943-019-0966-6

**Published:** 2019-03-30

**Authors:** Manendra Babu Lankadasari, Pramiti Mukhopadhyay, Sabira Mohammed, Kuzhuvelil B. Harikumar

**Affiliations:** 1 Cancer Research Program, Rajiv Gandhi Centre for Biotechnology (RGCB), Thiruvananthapuram, Kerala State 695014 India; 2 Manipal Academy of Higher Education (MAHE), Manipal, India; 3 Present address: Graduate School of Biomedical Sciences, University of Texas Health Science Center, San Antonio, TX 78229 USA

**Keywords:** Tumor-associated macrophages, Pancreatic cancer, Desmoplasia, Immunotherapy, Clinical trials, Stroma, Microenvironment

## Abstract

Among all the deadly cancers, pancreatic cancer ranks seventh in mortality. The absence of any grave symptoms coupled with the unavailability of early prognostic and diagnostic markers make the disease incurable in most of the cases. This leads to a late diagnosis, where the disease would have aggravated and thus, incurable. Only around 20% of the cases present the early disease diagnosis. Surgical resection is the prime option available for curative local disease but in the case of advanced cancer, chemotherapy is the standard treatment modality although the patients end up with drug resistance and severe side effects. Desmoplasia plays a very important role in chemoresistance associated with pancreatic cancer and consists of a thick scar tissue around the tumor comprised of different cell populations. The interplay between this heterogenous population in the tumor microenvironment results in sustained tumor growth and metastasis. Accumulating evidences expose the crucial role played by the tumor-associated macrophages in pancreatic cancer and this review briefly presents the origin from their parent lineage and the importance in maintaining tumor hallmarks. Finally we have tried to address their role in imparting chemoresistance and the therapeutic interventions leading to reduced tumor burden.

## Introduction

Pancreatic cancer is, by nature, an aggressive and deadly disease with mortality closely paralleling incidence. The relative 5 years survival rates are as low as 8%, which falls to 2% when diagnosed at a distant stage [1]. This is due to the status quo of patients being diagnosed when the cancer has metastasized, commonly to the liver, lung and/or peritoneum, coupled with the fact that the belligerent disease is rather resistant to chemo- and radiotherapies. Although risk factors such as smoking, obesity, family history, diabetes, life style, diet, lack of exercise, etc. have been identified as some of the factors predisposing to the disease, the exact causation is still to be elucidated [2–5]. The resistance to current therapies is conferred to the characteristically dense stroma associated with pancreatic tumors. Recent studies propose that the stiffness of the extracellular matrix stroma provides a hindrance to blood vessel perfusion and presents a barrier to drug delivery to cancer cells [6]. Pancreatic cancer is categorized into two types– tumors arising from the exocrine gland (adenocarcinoma, constitutes 95% of all pancreatic cancers) and from endocrine gland (often called islet cell tumors or neuroendocrine tumors, constitute 5%). Exocrine tumors tend to be more aggressive with poor prognosis and survival rate. Pancreatic Ductal Adenocarcinoma (PDAC) is the most common cancer, constituting nearly 90% of all pancreatic cancers. Surgical resection continues to be the only definitive treatment for PDAC, yet, the fraction of patients who have tumors that are amenable to surgical resection is only approximately 10–20% [7]. Molecular aspects of pancreatic cancers such as key genes responsible for driving cancer progression have been studied well and are still undergoing research [8–13]. Lack of clinical progress on pancreatic cancer when compared to other cancers slowed down the development of novel and effective therapies. The generation of a massive stromal tissue, which in some cases can make up to 80% of the tumor mass, is an archetypal feature of PDAC [14]. The tumor stroma of PDAC has both tumor suppressor and tumor promoting capacities [15, 16]. This concurs with the presence of a staggering milieu of cells in the tumor microenvironment (TME) such as regulatory T cells, immature monocytes, dendritic cells, mast cells, natural killer cells, neutrophils, cancer-associated fibroblasts (CAFs), pancreatic stellate cells and tumor-associated macrophages (TAMs). This heterogeneous population and its interactions with tumor stroma contribute to the ambivalence of the latter towards tumorigenesis. Contribution of the microenvironment to the tumor progression is essentially a novel perspective to identify new therapeutic targets. In this context, pancreatic cancer is known to have a dense complex stroma containing fibro-inflammatory mixture along with extracellular matrix, nerves and blood vessels. Recent studies already highlight the paradoxical roles of M2 macrophages and their distribution in the tumor architecture which determines the poor prognosis in pancreatic cancer [17]. Hence, immunotherapy coupled with molecular targeted therapy (e.g. against DNA repair genes) is a promising regimen which could yield improved outcomes rather than using relatively outdated cytotoxic drugs in pancreatic cancer [18].

### The origin of tumor-associated macrophages

The major immune cells associated with the stroma are the tumor-associated macrophages (TAMs), regulatory T cells, immature monocytes, mast cells, dendritic cells, natural killer cells and neutrophils. They accumulate in the tumor and collectively play myriad roles like immune suppression, tumor cell invasion and chemotherapeutic response. Among these, the major line of defense is drawn by the innate cells, macrophages, which participate in normal physiological roles like immune response, homeostasis and tissue repair with altered roles under diverse pathological conditions. They form the major tissue resident phagocytes which are important in host defense mechanisms and homeostasis [19]. Their involvement in a variety of malignancies makes them potent therapeutic targets. Recent studies in pancreatic cancer have raised skepticism about the immunological dogma for origin of TAMs from the circulating monocytes. On the contrary, it has now been proved that they are not always derived from hematopoietic stem cells and their origin is also from embryonic precursors seeded within the tissue with self-renewal capability. This reveals that the knowledge acquired regarding their origin is still not clear [20].

To further complicate this scenario, the macrophages exhibit functional plasticity to form either of two types of functionally dissimilar cells upon activation by specific polarization signals [21]. Based on their functions, they can be broadly categorized into two classes: classically activated M1 and alternatively activated M2 macrophages. TAMs resemble M2 macrophages and exhibit pro-tumor activity [22]. Various polarization signals like IFNγ and bacterial LPS activate M1 macrophages and once activated, they detect and clear tumor cells, pathogens and also present antigens to T cells. They are reservoirs of pro-inflammatory cytokines like IL-23, TNF, IL-6, and IL-12 and are involved in Th1 responses to infection. On the other hand IL-4, IL-13, IL-10 and TGF-β activate M2 macrophages, which in turn, produce IL-10 and IL-1b, which suppress Th1-mediated inflammation and induce a Th2 response. M2 macrophages promote wound healing and tissue remodelling by secreting matrix metalloproteinases which digest extracellular matrix. They can also exude vascular endothelial growth factor (VEGF) which promotes vascularisation [23]. Contradicting reports exist regarding the cellular origin of TAMs. A recent study by Franklin et al., disproved the already existing theories of M2 phenotype and posited that the TAMs are functionally and phenotypically divergent in comparison with traditional M2 macrophages. They highlighted the importance of Notch signalling in the differentiation of TAMs from inflammatory monocytes which express CCR2 [24]. A recent study exploited the usage of sex mismatched allogenic bone marrow transplants to track the origin of macrophages in the carcinoma of recipients. They found that the majority of CD163+ macrophages in the TME originated from the bone marrow [25].These findings further questioned our understanding of molecular origin of TAMs and highlighted the complexity and importance of further research.

### Polarization and metabolic reprogramming of macrophages

TME consists of immune cells (innate as well as adaptive) apart from the cancer cells as well as stroma. The coordinated communication between them ultimately determines tumor growth and progression. During normal physiological state, a sustained balance is maintained but, during tumorigenesis deregulated signalling in the microenvironment leads to the biased expression of various immune mediators (cytokines or chemokines). This leads to the activation/polarisation of specific phenotypes (antitumoral M1 or protumoral M2). Among the various immune cell types, the TAMs represent most of the leukocytic population with their well-documented roles in tumorigenesis and inflammation-driven cancers. Neoplastic cells release certain chemokines that recruit TAMs to the close vicinity of tumor cells. In return, TAMs facilitate tumor progression by providing supporting factors such as MMPs, cathepsins, VEGF, PDGF, FGF and various chemokines like CXCL8 that assist tumors to proliferate, invade and metastasize [26, 27].

During neoplastic transformation, the tumor cells start expressing C-C motif chemokine ligand 2 (CCL2), which is the major chemoattractant of monocytes and macrophages [28]. Apart from CCL2, there are certain other chemokine attractants exuded by the tumor mass such as CCL5, CXCL8, CCL7 and CXCL12 and a few other cytokines like VEGF and M-CSF [29]. The tumor milieu is already composed of a cocktail of cytokines and chemokines with no IFNγ or bacterial components. Upon arrival of these monocytes, these factors help in the maturation of the cells to become full-fledged M2-macrophages [30]. T-reg cells and fibroblasts secrete certain other factors like TGF-β and IL-10 which prop up this polarization. Another interesting aspect of this polarization cocktail is that TAMs, they themselves can secrete CCL2 which serve as an amplification loop. Here, TAMs perform varied flagrant functions like secreting tumor inducing factors like EGF, creating an immunosuppressive environment and promoting angiogenesis while also maintaining tumor related inflammation and inducing metastasis [31]. Although M1 macrophages show anti-tumorigenic property while M2 macrophages promote tumor progression, in light of recent studies, this binary belief has been disproved as TAMs have been demonstrated to show characteristics of both M1 and M2 macrophages, leaning more towards tumor promoting activity [32].

**Fig. 1:**
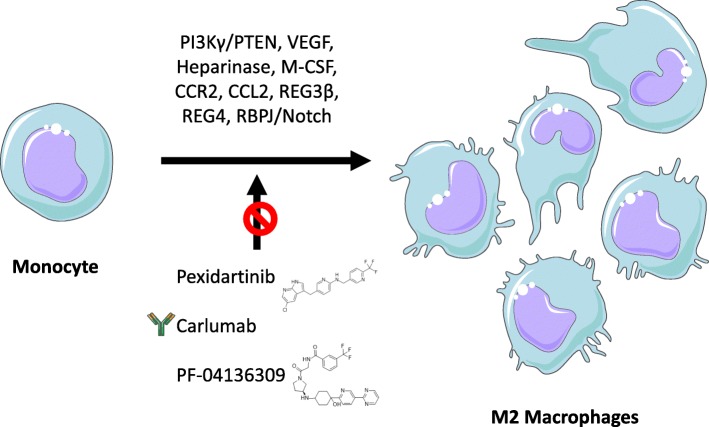
The emergence of M2 macrophages from monocytes. Various signaling molecules like M-CSF, CCL2 etc. and certain pathways like PI3Kγ/PTEN and RBPJ/Notch cascades are involved in M2 differentiation from monocytes. Therapeutic interventions like pexidartinib, carlumab and PF-04136309 can be used to block this differentiation

The TME drives the polarization of M2 macrophages in several ways. Cancer-associated fibroblasts infiltrating the tumor stroma in PDAC and induce M2 polarization through paracrine secretion of M-CSF. This is indicated by the blockade of M-CSF signalling markedly attenuating the generation of M2 macrophages [33, 34]. CSF1-dependent TAMs promote tumorigenesis and their infiltration correlates with the clinical aggressiveness of pancreatic neuroendocrine tumors (PNETs) [35]. Heparanase enzyme was revealed to promote lymphangiogenesis and tumor invasion in PNETs, where this enzyme produced by both TAMs and cancer cells is important for tumor progression [36]. A novel function of heparanase in molecular decision-making was proposed by Hermano et al., where in heparanase was indicated to influence cancer-promoting action of TAM. Examination of heparanase expression level hypothesizes relevance of the enzyme in defining a target patient subgroup that is likely to benefit the most from treatment modalities targeting TAM/IL-6/STAT3 [37]. The anti-inflammatory lectin REG3β is overexpressed in the serum and pancreatic juice of PDAC patients [38]. A recent study showed that REG3β inhibits M1 polarization while enhancing the M2 phenotype in an orthotopic mouse model of pancreatic cancer. REG3β deletion led to impaired vascularization, increase in apoptosis and a modified immunostroma composition [39]. REG4, another lectin, correlated with increased expression of MMP2 and MMP9 in pancreatic cancer [40]. REG4-induced EGFR/AKT/CREB signalling pathway is involved in macrophage polarization to M2 phenotype [41]. Hypoxia plays an important role in pancreatic cancer metastasis and a recent study delineated the role of exosomes in maintaining M2 phenotype during hypoxic conditions. They observed an enrichment of miR-301a-3p microRNA in the hypoxia emerged exosomes which resulted in the induction of M2 polarization via activation of PI3Kγ/PTEN signalling cascade resulting in hyper accumulation of M2 macrophages leading to metastasis [42]. The VCAM-1 in the pancreatic cancer cells induces Warburg effect by increased lactate production resulting in glycolytic phenotype in pancreatic tumors resulting in active TAM-like phenotype [43, 44] (Fig. 1).

TME is devoid of nutrients and TAMs should reprogram themselves to survive in such deprived conditions after polarization. The differences in M1 and M2 macrophages also includes a difference in metabolic preferences- M1 macrophages obtain energy through glycolysis while M2 macrophages have a relatively lower dependency on glycolysis and produce ATP mainly through the TCA cycle [45]. Metformin, an intervener in glucose metabolism, was reported to successfully reprogram TAMs towards an anti-tumoral phenotype in pancreatic cancer models [46]. A recent study suggests that the spatial patterning of TAM phenotypes is correlated with gradients of oxygen and lactic acid in the TME. TAMs expressing mannose receptor, C type 1 (MRC1) were found to be located in nutrient-rich regions while those expressing arginase 1 (ARG1) were spatially confined to hypoxic regions, indicating a gradient for vasculature morphogenesis in the TME [47]. The involvement of serotonin both in the dedifferentiation of acinar cells to duct-like progenitor cells [48] and in promotion of the Warburg effect in pancreatic cancer [49] provides an interesting aspect of exploration since M1 macrophages have been shown to be involved in the metaplasia of acinar cells [50].

### Roles of TAMS

#### TAMs in inflammation

Cancer-related inflammation, known as the seventh hallmark of cancer, is responsible for several tumor-promoting effects. TAMs act as an essential connecting moiety between inflammation and cancer via secretion of pro-inflammatory cytokines/chemokines. The M2 polarized macrophages, which constitute majority of the macrophages in TME, secrete IL10 and other cytokines that mediate (Th)-2 responses and are responsible for malignant tumor transformation and inhibit antitumor immune response mediated by T cells [51, 52]. Alternatively activated M2 macrophages suppress the adaptive immunity via secretion of cytokines for instance, high levels of IL-10, low levels of IL-12 and chemokines like CCL13, CCL18 [53].

Versican, a large ECM proteoglycan, activates Toll-like receptors, TLR-6 and TLR-2 on TAM which elicit the expression of inflammatory genes [54]. Activated TAMs produce IL-6 and TNF; in a mouse model of pancreatic cancer contributing to STAT3 activation. Ablation of IL-6 synthesis or STAT3 activation resulted in reduced carcinogenesis and inflammatory cell infiltration [55, 56]. TAMs are frequently found around pancreatic cancer cells but the contribution of these TAMs in malignant tumor progression and metastasis is not clear. A cell adhesion molecule VCAM-1 is reported to be overexpressed in PDAC tissues as well as in cell lines and is associated with clinical outcome of pancreatic cancer. VCAM-1 is strongly correlated with the CCL18, the most abundant inflammatory chemokine secreted by the TAMs. Recent report shows the effects of upregulated CCL18/PITPNM3/NF-kB/VCAM-1 signalling cascade in the pancreatic tumor progression [43].

#### TAMs in metastasis

Metastasis is an undesirable process associated with aggressive cancers that results in the development of detectable and often, incurable, tumors at sites distant from the original site of cancer. In general it starts with the production of CSF-1 from the tumor cells which induces EGF production in the TAMs. Both tumor cells and TAMs then move towards blood vessels to enter into blood stream. TAMs facilitate tumor cells to extravasate by increasing the permeability of the blood vessels via promotion of VEGF-A expression in tumor cells. Once tumor cells migrate and colonize the distant sites, they release CCL2 which further recruits inflammatory monocytes and convert them into metastasis-associated macrophages [57]. Pancreatic cancer is diagnosed in patients after metastases have formed at distant sites, which is the leading cause of pancreatic cancer-associated mortality. TAMs play a phenomenal role in promoting EMT. Coculturing of cancer cells with both PDAC-derived M1 and M2 macrophages contribute to promotion of metastasis with increased mesenchymal phenotype and markedly enhanced invasion through a collagen-I matrix, which was pronounced after co-culturing with M1 macrophage [58]. Hence, novel therapies targeting both M1 and M2 macrophages may have a better efficacy in lessening the metastasis of pancreatic cancer.

The exact mechanism by which TAMs affect this enhancement of metastasis is not clear, although a study by Penny et al indicated that TGF-β is the key factor in promotion of EMT by TAMs. Correlating with their pro-metastatic phenotype, TAMs favour glycolysis to fulfill their energetic needs and its inhibition can completely disrupt their pro-metastatic abilities [45]. In contrast to the above observation, a recent study showed an increased expression of TGF-β and IL-23 in the long term survivors in which they claim a reduced metastasis [59]. The macrophage inflammatory protein-3 alpha (MIP-3α) is a regulator of tumor cell invasion, produced by both TAMs and tumor cells [60]. MIP-3α, through its receptor CCR6, induce expression of MMP9 in pancreatic cells and thereby increase pancreatic cancer cell invasion through collagen Type IV [61]. CCR6 is also the receptor for the chemokine CCL20 which is produced by M2 macrophage, and mediates the effect of CCL20 on EMT and cell invasion of pancreatic cancer cells [62].

TAMs express the LPS receptor TLR4 on their surface, which has been implicated in a role in the EMT via TLR4/IL-10 cascade. Silencing of TLR4 or application of neutralizing antibodies against TLR4 and IL-10 separately showed a clear decline in proliferation induced by M2 macrophages. Coculturing of M2 macrophages with Panc1 and BxPC-3 cells also showed significantly decreased activity of MMP2 and MMP9 [63]. The conditioned media from pancreatic cancer cells increased M2 phenotype in THP-1 cells, inturn resulting in increased IL-8 production from TAMs leading to invasion [64]. The PI3Kγ/PTEN pathway in macrophages, induced during hypoxic state, also promotes metastasis [42].

#### TAMs in angiogenesis

Angiogenesis is one of the crucial events essential for the sustained growth and invasion of tumors. Cancer cells induce angiogenesis to escape from hypoxia and nutrient deprivation. TAMs are indeed reported in these hypoxic areas and their numbers closely associate with the blood vessels within tumors. TAMs express HIF-1α which acts as transcription factor for many of the angiogenic responsive genes such as VEGF, TNF-α, IL-1β, IL-8, PDGF, bFGF, thymidine phosphorylase and MMPs [65]. Macrophage depletion by creating null mutation of CSF-1 gene attenuated the angiogenesis switch suggesting their contributory roles in blood vessel formation [66]. VEGF-A has been recognized as one of the major pro-angiogenic cytokines released by TAMs. VEGF recruits macrophages into tumors, with a recent study showing that VEGF-stimulated migration of TAMs require VEGFR2 expressed by the macrophages and selective inhibition of VEGFR2 was shown to reduce recruitment of macrophages into orthotopic pancreatic tumors [67]. Emerging reports suggest the likely existence of a novel subpopulation of monocytes, which differentiate into angiogenic TAMs in cancers, probably also in pancreatic cancer, which needs to be validated [68].

PDAC tumors cause the creation of a hypoxic TME, since the rapidly dividing malignant cells quickly exhaust the available nutrients and oxygen that can be provided by the established vasculature. TAMs localize in this hypoxic TME and promote the expression of HIF-1 and HIF-2. Through the HIF-1 pathway, TAMs can induce VEGF-A through production of TGFβ and NRF2 activation [69]. Vasohibin-1, an intrinsic angiogenesis inhibitor, has been revealed to be regulated by the TGF-β/BMP signalling between TAM and pancreatic cancer cells [70]. TAMs also produce several metalloproteases, of which MMP9 may have complex effects beyond matrix degradation like promotion of the angiogenesis.

#### TAMs in immune evasion

The cross-talk between immune cells and cancer cells has been well documented identifying it as one of the hallmarks of the cancer [71]. Immune system plays a key role in clearance of malignant cells; however tumors have developed numerous strategies to evade the immune system by creating immunosuppressive niche. Unlike other cancer types such as melanoma or lymphoma very little is known about the strategies in PDAC that help it to overcome the immune system. Due to this strong coordination between cytokines and receptor-ligand pathways between tumors and stroma, even checkpoint monotherapies have failed in PDAC. Hence there is an urgent need to understand this rigid microenvironment in PDAC. The secretion of diverse immunosuppressive cytokines like IL10 by the TAMs helps in immune evasion. In the microenvironment, TAMs customise T cell responses through the induction of tolerogenic forkhead box P3 (FOXP3+) and IL 10 secreting T cells and also by upregulation of inhibitory receptor cytotoxic T-lymphocyte antigen 4 (CTLA-4) in autologous T cells [72]. Treg cells infiltrating the tumor stroma in pancreatic cancer express high levels of PD-1 and CTLA-4. Hence, blockade of the CTLA-4 and PD-1 pathway may enhance anticancer immune response by diminishing the number and suppressive activity of these intratumoral suppressor cells [73, 74].

FOXP3 is essential for the survival and activation of Treg cells, and is expressed by PDAC, inhibiting T cell activation. In a mutant KRAS model of pancreatic cancer, Tregs were localised in higher amounts within the TME early in disease progression [75]. TAMs which are positive for CD120a, CD120b can induce apoptosis in activated T cells in vitro and in vivo when they come in contact with them by secreting NO thereby eliminating the antitumor T cells [76]. The expression pattern of tumor cells is altered by TAMs so as to evade the T cell response, is yet another mechanism. This is done by the TAMs through induction of B7-homolog 4 (B7-H4) expression on the surface of the cancer cells in EGFR/MAPK dependent manner [77]. The cancer cells which over express B7-H4 could escape the T cell-tumor cell interaction resulting in inhibition of CD8+ T cells antitumor activity. TAMs also secrete various bioactive lipids like 15(S)-hydroxyeicosatetraenoic acid which has potential immunosuppressive functions [78]. The expression of arginase I by the TAMs plays an important role in immune surveillance by depleting the local L-arginine in the confined environment altering the T cell receptor expression and proliferation [79]. It is well known that glucose depletion blocks the function of anti-tumor T-cells [80] and high levels of lactate govern the polarization of macrophages [44]. The pancreatic tumors comprise of hypovasculature resulting in low glucose levels resulting in reduced antitumor T-cell activity.

#### TAMs in chemotherapy resistance

**Fig. 2:**
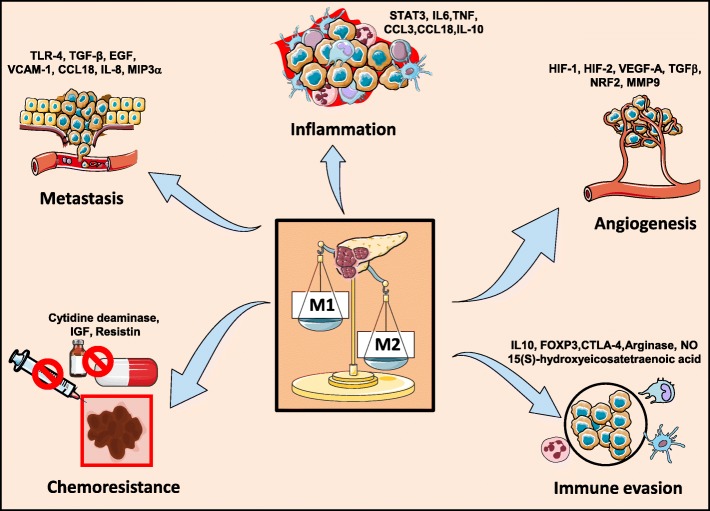
The role of TAMS in promoting pancreatic cancer. TAMs promote pancreatic cancer by modulating several key mechanisms in the body. These deregulations are involved in promoting inflammation, metastasis, angiogenesis, immune evasion and chemoresistance thereby leading to the aggressiveness of pancreatic cancer

Cancer cells develop chemoresistance by adopting various mechanisms during long-term exposure to chemotherapy. Tumor cell autonomous responses such as epigenetic alterations, activation/repression of survival/cell death pathways through drug inactivation, epithelial-mesenchymal transition and clonal selection for resistant population are only a few of the numerous mechanisms adapted by them [81]. A high incidence of drug resistance is reported in case of tumors with dense rigid stroma containing non-malignant cocktail of cells. Among these, TAMs have intricate signalling cross-talks with tumor cells which regulate the delivery of therapeutic drugs to the tumor sites and there by imparting resistance [82]. PDAC have acidic and distinctly dense stroma surrounding the tumor causing high solid stress and fluid pressure in the tumors that compress the vasculature, which, along with the architectural constraints is responsible for the hypovascularization [83]. TAMs can also mediate reaffirmation of the inherent resistance of PDAC. However, targeting TAMs alone is not sufficient to overcome the chemotherapy resistance since it is reported that an alternative compensatory influx of other myeloid subsets (TANs, Tumor-associated neutrophils) maintain the immunosuppressive TME recapitulating chemotherapeutic resistance. Targeting both CCR2^+^ macrophages (TAMs) and CXCR2^+^ neutrophils (TANs) together enhances the immunity and suppress therapeutic resistance [84]. TAMs induce upregulation of cytidine deaminase, the enzyme that facilitates the metabolism of gemcitabine upon its entry into the cell. Treatment of a transgenic PDAC mouse resistant to gemcitabine with GW2580, a CSF1-receptor antagonist, reduced TAMs and improved gemcitabine sensitivity on the PDAC tumors [85]. TAMs and myofibroblasts are the principal sources of insulin like growth factor (IGF) in the TME and this activates IGF1 receptor on pancreatic tumor cells resulting in enhanced chemoresistance [86]. A recent study showed that the drug Simvastatin induced Growth Factor Independent 1 Transcriptional Repressor (GFI-1) expression induced sensitivity towards gemcitabine in PDAC cells by depleting TGF-β1 secretion by TAMs, thereby attenuating TAM-mediated resistance to gemcitabine [87]. Peritoneal dissemination during metastasis is seen in pancreatic cancer patients and the presence of macrophages in close proximity with the peritoneal cancer cells was observed. These macrophages induce EMT in pancreatic cancer leading to drug resistant mesenchymal phenotype [88]. TAMs also secrete resistin, a ligand for CAP-1 and TLR-4 which upon interaction in tumor cells lead to activation of STAT3 and thereby gemcitabine resistance [89] (Fig. 2).

#### TAMing of pancreatic cancer: Therapeutic potential of TAMs

TAMs play an important role in multiple stages of tumorigenesis such as tumor initiation, inflammation, immune evasion progression and metastasis, angiogenesis and chemoresistance of pancreatic cancer, thereby making them an ideal and attractive therapeutic target (Fig. 2). After the success of alemtuzumab, nivolumab and ipilimumab, immunotherapy is gaining more attention in cancer therapy as the conventional therapy saw limited success for decades, much of which resulted in drug resistance. Core research is focused on oncovaccines, blocking immune check point cascade, antibodies against cancer antigens or by stimulating adaptive immune response. Since M2-type TAMs along with cancer cells possess CTLA-4, PD1 and PDL1 ligands, targeting TAMs stand as a promising immunotherapeutic approach to treat cancer [90]. TAMs have been described to have a yin-yang effect on tumorigenesis and can be concomitant with anti-cancer therapies in more ways than one [91].

#### Reducing the number of TAMs

Findings from the past decade showed that the aggressiveness of the tumor correlated with the localization and number of TAMs. Therefore, many ideal strategies to reduce the number of TAMs in the tumor architecture are being developed. Inflammatory monocytes expressing CCR2 are recruited into the stroma by the expression of its chemokine ligand CCL2 and matured to form TAMs. Blockade of CCL2 leads to an enhanced expression of M1 polarization-associated genes and cytokines, along with reduced expression of M2-associated markers in human macrophages [92]. Carlumab, monoclonal antibody directed against CCL2, was tested in pancreatic cancer patients but needs further studies to assess its impact on macrophages, as the number of patients enrolled in the trial were less [93]. Further Targeting C-C motif chemokine receptor 2 (CCR2) or colony stimulating factor 1 receptor (CSF1R) rallies chemotherapeutic efficacy inhibits metastasis and increases anti-tumor T-cell responses by causing a reduction in the number of tumor-initiating cells (TICs) in PDAC [94]. IL-27, an interleukin with potent anti-tumor effects, is primarily produced by activated antigen presenting cells (APC) including macrophages and dendritic cells (DCs) [95]. IL-27 inhibited proliferation, migration and invasion of pancreatic cells and induced apoptosis when co-cultured with M2 polarized macrophages. It also enhanced the efficacy of gemcitabine by targeting TAMs, which could provide a front for a novel therapy to target macrophages to reduce the aggressive behavior of pancreatic cancer cells [96]. The CCL2/CCR2 axis has prognostic significance in pancreatic cancer and was suggested as an effective immunotherapeutic target for a novel CCR2 inhibitor (PF-04136309). In an orthotopic model of murine PC, it was seen that inhibition of CCR2 by PF-04136309 promoted anti-tumor immunity [97]. A single-centre, open-label, dose-finding, non-randomised, Phase 1b trial was conducted to study the effect of CCR2-targeted therapy with PF-04136309 in combination with FOLFIRINOX, a chemotherapy regimen for treatment of metastatic pancreatic cancer and proved it to be safe and tolerable [98]. The study was further extended to target TAMs and TANs using CCR2i and CXCR2i respectively and showed an improved response in orthotopic PDAC tumors with FOLFIRINOX. Further investigations and trials are needed to determine the efficacy of this proposed therapy [84].

#### Redirecting TAMs

Polarization of macrophages plays an important role in converting monocytes to M2 macrophages and vice versa. The conversion of M2 phenotype to M1 phenotype may thus result in improved immunosurveillance. Signal regulatory protein-a (SIRPα), a molecule in macrophages, is bound by its transmembrane protein ligand CD47, resulting in the inhibition of engulfment by macrophages through a signalling cascade mediated via phosphorylation of the immunoreceptor tyrosine-based inhibitory motif on the cytoplasmic tail of SIRPα [99, 100]. CD47 is expressed by PDAC tumor cells, including the cancer stem cells (CSCs). Cioffi et al. demonstrated that inhibition of CD47 with anti-CD47 mAbs may offer a new opportunity to turn TAMs against PDAC cells, including CSCs, by activating phagocytosis. This in combination with gemcitabine, would allow the resistance of PDAC against chemotherapy to be overcome [101]. LPS triggers a close physical proximity between CD14, and TLR4 [102]. Priming of TAMs with TLR4 agonist (LPS) alone or in combination with IFN-γ altered M2 polarization towards M1 and also induced a strong anti-cancer immune reaction [103].

An unanticipated role of nab-paclitaxel has been observed where macropinocytosis of nab-paclitaxel by TAMs causes a switching to M1 polarization and increased expression of cytokines by M1 macrophages via TLR4, both in vitro and in orthotopic model of PDAC [104, 105]. This revealed that the mechanism is similar to that of paclitaxel, which stimulates M1 polarization by acting as an LPS mimetic [106]. CD40, a member of the tumor necrosis factor receptor (TNFR) family, and its ligation have been shown to have anti-tumor effect via agonistic anti-CD40 mAbs to either directly kill CD40-positive tumor cells or activate T-cell immune responses [107, 108]. Beatty et al. demonstrated the efficacy of using CD40 agonists in combination with gemcitabine therapy by altering the tumor stroma in PDAC, effecting a T cell anti-tumor activity and recalibration of TAMs to become tumoricidal [109]. This re-education of TAMs occurs via inhibition of the NF-κB pathway [110]. It may also involve IFN-γ and CCL2, as demonstrated by Long et al., [111].

Histidine-rich glycoprotein (HRG), produced by macrophages and megakaryocytes, is a host-produced antiangiogenic and immunomodulatory factor, which links thrombospondins (TSP), heparin, Fcγ receptors (FcγR) and other molecules that have been implicated in tumorigenesis. It has been reported to skew TAM polarization away from the M2- to a tumor-inhibiting M1-phenotype, thereby promoting anti-tumor immune responses and vessel normalization [112]. Pharmacological inhibition of macrophage lipid kinase, PI3kγ leads to reprogramming of TAMs to M1 resulting in restoration of CD8+ T cell mediated tumor suppression, reduced desmoplasia, metastasis and improved chemotherapeutic response [113]. Recent studies on IL27 revealed a contrasting unexpected function of this JAK-STAT pathway activator. The conversion of M2 to M1 phenotype of macrophage resulting in reduced proliferation and improved gemcitabine sensitivity was achieved by eliciting TAMs with IL27 [96]. An adjuvant therapy involving activation of RIG-I by its agonist showed enrichment in the targeted delivery using nanoparticle encapsulation. The amalgamation of RIG-1 agonist with BCL2 siRNA showed a strong induction in Th1 immune response and higher M1 macrophages with reduced immunosuppressive plasma and B cells [114]. Certain small molecules from natural sources like osthole from *Cnidium monnieri* [115] and urolithin A from *Punica granatum* [116] are also emerging as immunomodulators which majorly influence M2 polarisation in PDAC.

### Clinical trails

Translating the observations and conclusions to the betterment of human lives should be the ultimate goal that drives a research. Prior to marketing a drug, the efficiency and toxicity should be well documented through clinical trials. Understanding the origin, recruitment and polarisation of TAMs has brought out many signaling pathways important in their establishment inside tumor. For instance, CCL2, VEGF, M-CSF etc. secreted by the tumor stroma, attract and recruit the circulating monocytes into the microenvironment [29]. The monocytes are polarised into M2 macrophages by priming with M-CSF, IL3, IL4, IL-10, TGF-β etc. [23]. By blocking the above pathways using specific inhibitors or antibodies, the formation of TAMs can be inhibited. Many ongoing clinical trials target M-CSFR, VEGFR, PI3K and the outcome will emphasize the importance of TAMs in pancreatic cancer. Other important inhibitors like bindarit (CCL2 inhibitor), OMP-21 M18 (antibody against D114, Notch pathway inhibitor), LY364947 (TGF-β/SMAD inhibitor) SL-501 (IL3R inhibitor), pascolizumab (IL4R inhibitor) etc. should be tested in combination with standard cytotoxic drugs to check their efficiency. Several clinical trials from past two years have been tried out targeting TAMs with the hope of finding a cure for pancreatic cancer (Table 1).

**Table 1:** The ongoing clinical trials of past two years which targets TAMs in pancreatic cancer

S. No	Curative Agent	Mode of Action	ClinicalTrial.gov ID	Phase	Expected Outcome (with regard to TAMs)
1.	Nab-paclitaxel + Gemcitabine + OMP-59R5	Anti-Notch2/3 Antibody, macropinocytosis	NCT01647828	II	↓ M2 origin and polarization
2.	Olaparib + Cediranib	PARP and VEGFR inhibitor	NCT02498613	II	↓ M2 recruitment
3.	CRS-207 + GVAX ± Nivolumab	Irradiated GM-CSF, listeria antigen, Anti-PD-1 antibody	NCT02243371	II	↑ Macrophage count
4.	Sorafenib + Gemcitabine + Vorinostat + Radiotherapy	VEGFR, PDGFR inhibitor HDAC inhibitor	NCT02349867	I	↓M2 recruitment
5.	Pexidartinib + Durvalumab	CSF-1R, Kit and Flt3 inhibitor, anti-PD-1 antibody	NCT02777710	I	↓ M2 polarization
6.	Pembrolizumab + AMG820	Anti-PD-1 antibody, anti-CSF1R antibody	NCT02713529	II	↓ M2 polarization
7.	Gemcitabine/Nabpaclitaxel + MM141	Bispecific Her3 and IGF antibody, macropinocytosis	NCT02399137	II	↓ M2 polarization
8.	Galunisertib + Durvalumab	TGF-β receptor inhibitor, anti-PD-L1 antibody	NCT02734160	I	↓ M2 differentiation
9.	Buparlisib + mFOLFOX6	PI3K inhibitor	NCT01571024	I	↓ M2 polarization
10.	GVAX + Cyclophosphamide + CRS-207	Listeria antigen, irradiated GM-CSF	NCT01417000	II	↑ Macrophage count
11.	Gemcitabine/Capecitabine + LY3023414 + Abemaciclib	PI3K/DNA-PK/mTOR inhibitor CDK inhibitor	NCT02981342	II	↓ M2 polarization
12.	Nivolumab + Cabiralizumab	Anti-PD-1 antibody, anti-CSF1R antibody	NCT03599362	II	↓ M2 polarization and recruitment
13.	Nivolumab + cabiralizumab + gemcitabine	Anti-PD-1 antibody, anti-CSF1R antibody	NCT03697564	IV	↓ M2 polarization and recruitment
14.	Cyclophosphamide + GVAX + pembrolizumab + IMC-CS4	Listeria antigen,Anti-PD-1 antibody	NCT03153410	I	↑ Macrophage count and ↓ M2 polarization
15.	5-fluorouracil + bevacizumab + leucovorin + oxaliplatin	Anti-VEGF antibody	NCT03127124	Ib	↓ M2 recruitment and differentiation
16.	Capecitabine + temozolomide +/- Bevacizumab	Anti-VEGF antibody	NCT03351296	II	↓ M2 recruitment and differentiation
17.	Palbociclib + Gedatolisib	PI3K/mTOR inhibitor	NCT03065062	I	↓ M2 polarization
18.	Cabiralizumab + nivolumab + gemcitabine + Nabpaclitaxel	Anti-PD-1 antibody, macropinocytosis, anti-CSF1R antibody	NCT03336216	II	↓ M2 polarization and recruitment

The observation of their outcomes will confirm the clear involvement of TAMs. The expected outcome based on the preclinical literature available is presented in the last column. The ↑ and ↓ signifies the increased and decreased respectively with the context.

## Conclusion

The morbid prognosis of patients diagnosed with PDAC demands effective therapeutic strategies against the aggressive metastatic character of the cancer. This review attempts to put forward a comprehensive collation regarding the therapeutic potential of tumor-associated macrophages in pancreatic cancer. The various strategies outlined herein provide an insight into the ongoing research in utilizing the pro-tumor characteristic of TAMs and the ability to re-educate these cells to create novel therapeutic interventions for PDAC patients. Given the dense network of cell signaling that is dysregulated in cancer cells, it is likely that further potential targets involved in the function of TAMs can be revealed. M2 TAMs have been shown to be associated with YAP1 signaling, which correlated with tumorigenesis in several cancer types [117], and recent studies have discovered the involvement of YAP1/HIF-α pathway in promoting cancer stem cells in pancreatic cancer [118]. The mTOR signaling pathway has become one of the most studied pathways due to its diverse functions. Wenes et al.*,* established a functional link between the ability of TAMs in promoting angiogenesis and the cellular metabolism in TAMs by downregulation of the mTOR inhibitor REDD1 in these cells. REDD1-deficient TAMs outcompete normal cells in terms of glycolysis and form more vascular junctions [119]. Although TAM populations in tumor stroma are high, marking them as a probable prognostic factor, the multiple roles that TAMs play in pancreatic cancer progression have not yet been delineated. *Franklin* et al.*,* already showed the importance of Notch signaling in monocyte differentiation into TAMs [24]. Additional mechanistic insight into the pathways which regulate the differentiation of TAMs from monocytes is required. The induction of DNA damage caused by TAMs is still unknown and further studies have to be done on the role of TAMs in genomic instability. A strong correlation does exist; as inflammation induced NF-κB activates the activation-induced cytidinedeaminase (AID) which is the key DNA mutating enzyme. Its expression is also very important during E-M transition. TAMs play key roles during all these steps and so the link between AID and TAMs should be elucidated. Further studies into both upstream and downstream regulators of TAMs besides the cells themselves therefore comprise an appreciable source of potential therapeutic targets for pancreatic cancer. Though a large number of clinical trials on pancreatic cancer patients are being carried out worldwide, the focus is not always on TAMs. The majority of the clinical trials are based on PD1 therapy to activate Th1 response but the ultimate cause of this suppression, i.e., TAMs has not been aimed. A need for meaningful combinatorial studies which could block M2 polarisation with increased T cell response and induced cytotoxicity towards cancer cells should emerge to halt this epidemiological crisis.

## Abbreviations

AKT: Ak strain transforming
BCL2: B cell lymphoma 2
BMP: Bone morphogenetic protein
CAP-1: Adenylate cyclase-associated protein 1
CCR: C-C Chemokine Receptor
CD: Cluster of Differentiation
CREB: cAMP response element-binding protein
CXCL: chemokine (C-X-C motif) ligand
ECM: *Extracellular matrix*
EGFR: Epidermal growth factor receptor
FOLFIRINOX: FOLinic acid Fluorouracil IRINotecan OXaliplatin
HIF1: Hypoxia Inducible Factor 1
IFNγ: Interferon gamma
IL: Interleukin
JAK: Janus kinase
LPS: Lipopolysaccharide
MAPK: Mitogen- activated protein kinase
M-CSF: Macrophage colony-stimulating factor
MMP: Matrix metalloproteinases
mTOR: Mammalian target of rapamycin
NF-kB: Nuclear factor kappa-light-chain-enhancer of activated B cells
NO: Nitric Oxide
NRF2: Nuclear factor erythroid 2–related factor 2
PD-1: Programmed Cell Death 1
PDL-1: Programmed death-ligand 1
PI3Kγ: Phosphatidylinositol-4,5-bisphosphate 3-kinase gamma
*PITPNM*: Phosphatidylinositol Transfer Protein Membrane Associated
PTEN: Phosphatase and tensin homolog
REG: Regenerating *gene*
RIG-I: *Retinoic* Acid Inducible *Gene* I
siRNA: Small interfering RNA
STAT: Signal transducer and activator of transcription
TAM: Tumor-associated macrophage
TAN: Tumor-associated neutrophils
TGF: Transforming growth factor
Th: T helper
TNF: Tumor Necrosis Factor
VCAM: Vascular cell adhesion protein
YAP1: Yes-associated protein 1
